# Transcriptional response to ^131^I exposure of rat thyroid gland

**DOI:** 10.1371/journal.pone.0171797

**Published:** 2017-02-21

**Authors:** Nils Rudqvist, Johan Spetz, Emil Schüler, Toshima Z. Parris, Britta Langen, Khalil Helou, Eva Forssell-Aronsson

**Affiliations:** 1 Departments of Radiation Physics, Institute of Clinical Sciences, Sahlgrenska Cancer Center, Sahlgrenska Academy, University of Gothenburg, Gothenburg, Sweden; 2 Departments of Oncology, Institute of Clinical Sciences, Sahlgrenska Cancer Center, Sahlgrenska Academy, University of Gothenburg, Gothenburg, Sweden; Van Andel Institute, UNITED STATES

## Abstract

Humans are exposed to ^131^I in medical diagnostics and treatment but also from nuclear accidents, and better knowledge of the molecular response in thyroid is needed. The aim of the study was to examine the transcriptional response in thyroid tissue 24 h after ^131^I administration in rats. The exposure levels were chosen to simulate both the clinical situation and the case of nuclear fallout. Thirty-six male rats were i.v. injected with 0–4700 kBq ^131^I, and killed at 24 h after injection (D_thyroid_ = 0.0058–3.0 Gy). Total RNA was extracted from individual thyroid tissue samples and mRNA levels were determined using oligonucleotide microarray technique. Differentially expressed transcripts were determined using Nexus Expression 3.0. Hierarchical clustering was performed in the R statistical computing environment. Pathway analysis was performed using the Ingenuity Pathway Analysis tool and the Gene Ontology database. T4 and TSH plasma concentrations were measured using ELISA. Totally, 429 differentially regulated transcripts were identified. Downregulation of thyroid hormone biosynthesis associated genes (e.g. thyroglobulin, thyroid peroxidase, the sodium-iodine symporter) was identified in some groups, and an impact on thyroid function was supported by the pathway analysis. Recurring downregulation of *Dbp* and *Slc47a2* was found. *Dbp* exhibited a pattern with monotonous reduction of downregulation with absorbed dose at 0.0058–0.22 Gy. T4 plasma levels were increased and decreased in rats whose thyroids were exposed to 0.057 and 0.22 Gy, respectively. Different amounts of injected ^131^I gave distinct transcriptional responses in the rat thyroid. Transcriptional response related to thyroid function and changes in T4 plasma levels were found already at very low absorbed doses to thyroid.

## Introduction

The beta particle emitter ^131^I is one of the most common radionuclides in nuclear medicine. ^131^I is administered as halide to treat hyperthyroidism and thyroid cancer, and may potentially be used to treat other tumors expressing the sodium iodide symporter (NIS, the transporter of iodide into the thyroid follicular cells) [1–3]. ^131^I is also bound to various tumor targeting molecules and administered systemically [4–7]. Treatments demanding administration of ^131^I as halide may result in accumulation of ^131^I in and irradiation of normal thyroid tissue (if present). Additionally, unbound and released (e.g. from *in vivo* metabolization of ^131^I-labeled pharmaceuticals) ^131^I may accumulate in and irradiate the normal thyroid [4, 8, 9]. Uptake of ^131^I in the thyroid can be partially blocked using e.g. potassium iodide. European Association of Nuclear Medicine (EANM) guidelines for ^131^I-MIBG (metaiodobenzyl guanidine) therapy report an absorbed dose of 0.05 Gy per GBq to the blocked thyroid, but higher absorbed doses have also been published, and administration of 1.9–11 GBq ^131^I-MIBG resulted in 0.2–30 Gy to the blocked thyroid [6, 10]. Normal thyroid may also be exposed to ^131^I from nuclear fallout and approximately 1,760,000 and 100,000–200,000 TBq of ^131^I were released in conjunction with the Chernobyl and Fukushima nuclear power plant accidents, respectively [11–13]. Median absorbed doses to thyroid of 356 and 39 mGy been reported in Belarus and Russia, respectively [14]. The risk of carcinogenesis after ^131^I therapy of e.g. hyperthyroidism is not clear [15], although accidental exposure of thyroid due to ^131^I fallout has been correlated with increased risk of thyroid cancer in children already after 200 mGy [12, 14]. Additionally, external radiation-induced thyroid tissue carcinogenesis has been established with a linear relationship between absorbed dose and incidence for absorbed doses as low as 100 mGy [16]. There are gaps in knowledge of ^131^I-induced effects in thyroid tissue, especially concerning the mechanisms involved. Therefore, it is necessary to identify molecular events that occur in response to ^131^I exposure in the normal thyroid gland.

The RNA microarray technique enables high-throughput analysis of global transcriptional changes between two or more samples. The result is a transcriptional profile that can be used to assess changes in cellular activity and biological functions, prediction of upstream/downstream regulation of target molecules, and for biomarker identification. We have performed several studies showing the effects of ^131^I and ^211^At on transcriptional responses in mouse thyroid gland, in non-thyroidal mouse tissues, and on the systemic relation between thyroid and non-thyroid tissues, as well as in mouse kidney after ^177^LuCl_3_ and ^177^Lu-octreotate administration [17–25]. Those studies were all performed in mice, where pooled thyroid samples often were used to obtain large enough amounts of tissue for analyses.

The aim of the present study was to identify transcripts involved in the acute biological response to ^131^I in rat thyroid tissue and to assess ^131^I-induced effects on thyroid function. The study was performed in normal rats, both to enable analyses of individual thyroid glands for better statistics, and also to examine if similar results were obtained in rats as previously found in nude mice.

## Material and methods

### Radionuclides and radioactivity measurements

^131^I was purchased as Na^131^I from GE Healthcare (Braunschweig, Germany). A gamma counter (Wallac 1480 Wizard® 3"; Wallac Oy, Turku, Finland) was used for ^131^I activity measurements of aliquots of the stem solutions to determine injected activities.

### Animal experiments

Thirty-six normal and healthy adult male Sprague Dawley rats (Scanbur AB, Sollentuna, Sweden) were randomly divided into nine groups (n = 4, to enable statistical calculations) and i.v. injected with 150 μl saline containing 0, 9.0, 88, 170, 260, 340, 760, 1300, or 4700 kBq ^131^I in the tail vein. Rats were injected in the order described in the previous sentence, with the exception of the control animals, which were mock-treated after the 4700 kBq group. The rats had access to water and standard laboratory chow *ad libitum*. All rats were well during the study time. Twenty-four hours after administration, animals were killed by cardiac puncture under anesthesia with sodium pentobarbital (APL, Sweden). Thyroids were then excised and stored at -80°C until RNA extraction. The experiment was performed during day-time in a dedicated animal laboratory (lights on during day-time). Rats were kept in standard laboratory rat cages with 4 rats in each cage. The study design was approved by the Ethical Committee on Animal Experiments in Gothenburg, Sweden (Permit Number: 166–2011).

### Estimation of absorbed dose

The mean absorbed dose to thyroid was estimated based on previously reported value of 6.1 Gy/MBq 24h after ^131^I injections in Sprague Dawley rats eating iodine-deficient chow (0.05 ppm iodine) five days prior to administrations [26]. The rats in the present study however, were eating standard laboratory chow (2 ppm iodine). Previous results show that Sprague Dawley rats on standard laboratory chow have nine times lower ^131^I activity concentration in thyroid at 24 h compared with Sprague Dawley rats on iodine-deficient chow (standard laboratory chow: 36%IA/g, SEM = 4, n = 32, data not published) [26]. Assuming otherwise similar ^131^I biokinetics in both animal models, a value of 0.65 Gy/MBq after 24 h was used in the present investigation.

### Gene expression analysis

Extraction and quality control of RNA have been described elsewhere [17]. RNA from individual thyroid tissue samples was processed at the Bioinformatics and Expression Analysis core facility at Karolinska Institute (Stockholm, Sweden) using Agilent SurePrint G3 Rat GE 8x60K (Agilent, Santa Clara, CA, USA). Nexus Expression 3.0 (BioDiscovery; El Segundo, CA, USA) was used to identify differentially expressed transcripts in exposed vs. non-exposed thyroids with fold change and FDR adjusted p-value cut-offs of 1.5 and 0.01 (Benjamini-Hochberg method), respectively. Herein, the term “regulated” is used synonymously to “statistically significantly differentially expressed”.

Hierarchical clustering using the complete linkage algorithm and Lance-Williams dissimilarity update formula [27] and heatmaps were created using the hclust and heatmap.2 functions (stats package verion 3.1.1 and gplots package, version 2.14.2, respectively) in the R statistical computing environment (http://www.r-project.org), respectively.

The output data from Nexus Expression 3.0 include functional annotation of genes using Gene Ontology (GO) terms. In the present investigation, genes with functional annotation to GO terms related to the thyroid are shown.

Gene expression data presented in this publication have been deposited at the NCBI’s Gene Expression Omnibus (GEO accession number GSE66623).

cDNA was prepared from total RNA isolated from thyroid using SuperScript® III First-Strand Synthesis SuperMix (ThermoFisher Scientific) for quantitative real-time RT-PCR (qPCR). qPCR reactions was then performed in triplicates for *Cdkn1a* (Rn00589996_m1), *Dbp* (Rn01498425_m1), *Slc5a5* (Rn00583900_m1), *Iyd* (Rn01430509_m1), *Pax8* (Rn00579743_m1), *Tg* (Rn00667257_g1), and *Tpo* (Rn00571159_m1) with Applied Biosystems 7500 Fast Real-Time PCR System using TaqMan Gene Expression Master Mix (ThermoFisher Scientific, initial activation at 50°C for 2 min, then 95°C for 10 min, followed by 40 cycles of 95°C for 15 s and 60°C for 1 min). The rationale behind selecting these genes for qPCR was as follows. *Cdkn1a*, also known as p21, plays a role in the p53 signaling pathway and was earlier shown to be induced by ionizing radiation [28]. *Dbp* was down-regulated with high consistency at lower absorbed dose levels in the present study, and was radiation-induced in a previous study on mice thyroid tissue exposed to ^131^I or ^211^At [29]. *Slc5a5*, *Iyd*, *Pax8*, *Tg*, and *Tpo* were selected because they are related to thyroid function, and are representative of interesting dose-response pattern with induction in only some animal groups seen in the present study. Delta CT (ΔCT) was calculated for each gene within each animal using the following formula: ΔCT(gene of interest) = CT(average of reference genes)–CT(gene of interest). *Tarbp2*, *Hprt1*, and *Tbp* were used as reference genes. Student’s t-test was used to test if averages between groups were statistically significant different (p-values are shown in figure). Due to low RNA yields, qPCR measurements were only performed for animals administered 0, 9.0, 88, 170, and 4700 kBq (0, 0.0058, 0.057, 0.11, and 3 Gy, respectively; n = 3/group). For animal-wise correlation of microarray and qPCR, linear regression analysis was performed. For qPCR, dCT values were used, and for microarray, normalized log_2_ intensity values were used. Goodness of fit was determined with R^2^ (calculated with Prism) and a p-value < 0.05 was used to determine if the slope was separate from 0.

### Upstream regulators, canonical pathways, and disease and function analyses

The Ingenuity Pathway Analysis tool (IPA, Ingenuity® Systems, www.ingenuity.com; Redwood City, CA) was used to assess upstream regulators, canonical pathways, and diseases and functions. Only endogenous upstream regulators were analyzed in the present study, e.g. drugs and chemical toxicants were disregarded during analysis. Upstream regulators were predicted to be activated and inhibited for z-scores ≥ 2 and ≤ -2, respectively. In the canonical pathway and disease and function analyses, a p-value cutoff of 0.05 was used (Fisher’s exact test).

### TSH and T4 plasma level analyses

Plasma concentration levels of thyroid stimulating hormone (TSH) and thyroxine (T4) were measured using ELISA kits. T4 plasma concentration levels were measured using the Thyroxine (T4) ELISA (mouse/rat) kit (Genway Biotech, Inc., San Diego, CA, USA) according to the manufacturer’s instructions. TSH plasma concentration levels were measured using the Rat Thyroid Stimulating Hormone (TSH) ELISA kit (Cusabio, Wuhan, Hubei Province 430206, China), according to the manufacturer’s instructions. However, due to insufficient plasma sample volume, a sample and standard volume of 50 instead of 100 μl was used. Additionally, also due to insufficient plasma volumes, TSH measurements of one and two animals administered 0, 170, 1300, and 4700 kBq, and 760 kBq, respectively, were only measured once, and one animal administered 260 kBq was not included in the TSH measurements. For both T4 and TSH measurements, Student’s t-test was used to determine statistical significance (p < 0.05).

## Results

### Dosimetry

Estimated thyroid absorbed doses were 0.0058, 0.057, 0.11, 0.17, 0.22, 0.50, 0.84, and 3 Gy for groups receiving 9, 88, 170, 260, 340, 760, 1300, and 4700 kBq, respectively.

### Regulated transcripts

**Fig 1:**
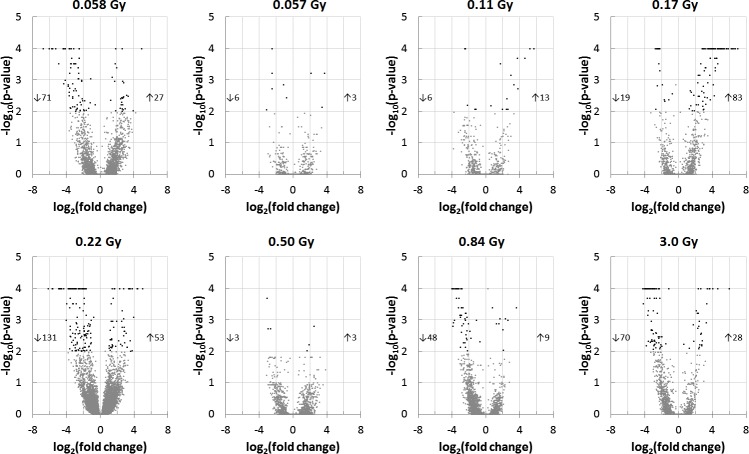
Volcano plots of regulated transcripts in rat thyroid exposed to 0.0058–3 Gy 24 h following ^131^I injection. Regulated transcripts with a fold change ≥ 1.5 (log_2_ ratio value ≥ 0.58) and Benjamini-Hochberg (False Discovery Rate) adjusted p-value < 0.01 are shown as black dots. Gray dots are transcripts not reaching the statistical cut off. The number of regulated transcripts can be seen for each group, e.g. 71 and 27 transcripts were down- and upregulated, respectively, in response to 0.0058 Gy. Adjusted p-values < 0.0001 are shown as 0.0001.

**Fig 2:**
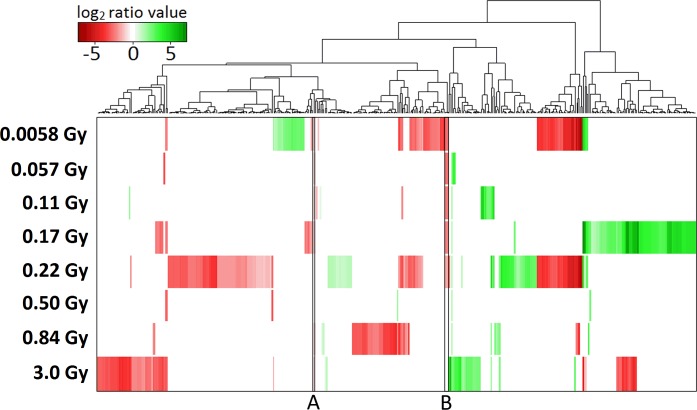
Hierarchical clustering of regulated transcripts in rat thyroid exposed to 0.0058–3 Gy 24 h following ^131^I injection. Regulated transcripts with a fold change ≥ 1.5 (log_2_ ratio value ≥ 0.58) and an adjusted p-value ≤ 0.01 were hierarchically clustered according to log_2_ ratio values using the hclust and heatmaps.2 functions in the R statistical environment. Statistically nonsignificant transcripts have been given the log_2_ ratio value 0. The transcript above “A”, downregulated in response to 0.0058–0.17 and 0.84–3.0 Gy, is the *Slc47a2* transcript. The transcripts above B are three *Dbp* transcripts, downregulated after 0.0058–0.22 Gy.

**Fig 3:**
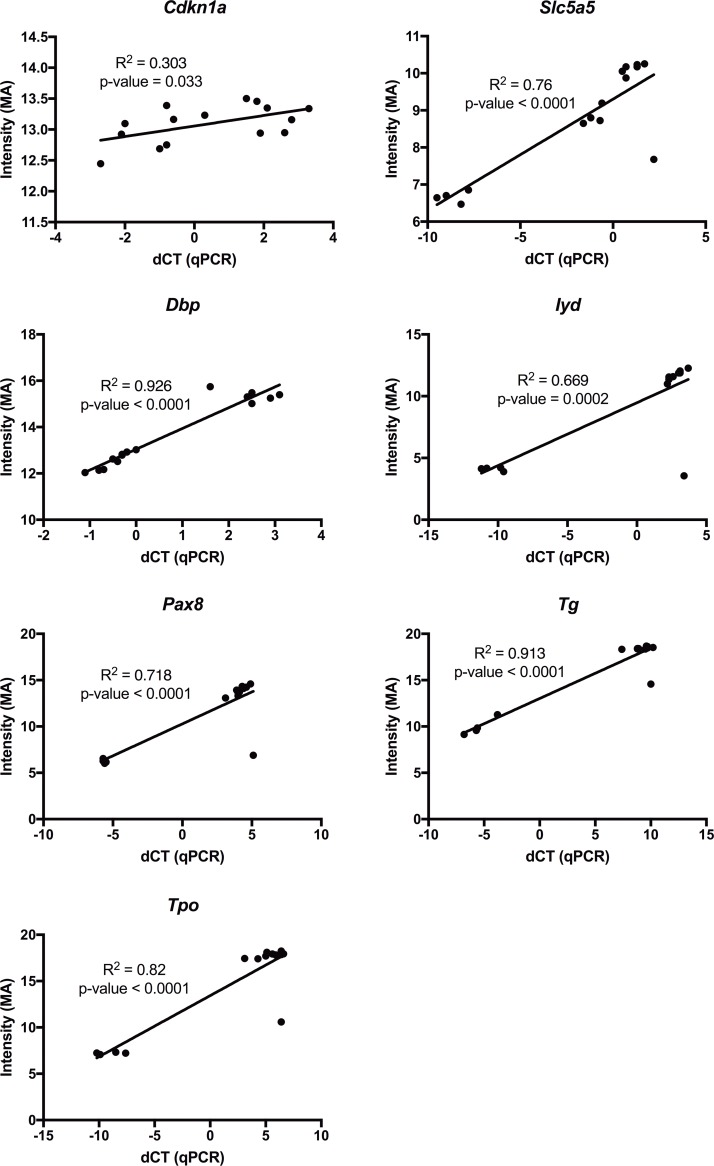
qPCR validation of microarray measurements for *Cdkn1a*, *Dbp*, *Slc5a5*, *Iyd*, *Pax8*, *Tg*, and *Tpo*. qPCR was used for validation of the microarray measurements. Scatter-plot x-axis: dCT values (calculated as CT_average of reference genes_−CT_gene of interest_); y-axis: normalized intensity microarray values. R^2^ (goodness of fit) and p-values (hypothesis: "Is slope significantly non-zero?") were calculated using Prism.

In total, 429 transcripts were regulated in rat thyroid glands 24 h after ^131^I administration (Fig 1, Table 1). Comparison log_2_ ratio values of all 30367 probes are shown in S1 Table. The number of regulated transcripts in each group varied from six (0.50 Gy) to 184 (0.22 Gy) (Fig 1, Table 1). Downregulation was prominent in all groups except in those exposed to 0.11, 0.17, and 0.50 Gy where the ratio between number of up- and downregulated transcripts were 2.2, 4.4, and 1, respectively. Regarding strength of regulation, the average fold change of regulated transcripts was lower in animals where the thyroid absorbed dose was 0.057, 0.50, and 0.84 Gy. There were also higher levels of upregulation compared with downregulation in response to 0.11 and 0.17 Gy, considering fold changes, while the opposite was found after 0.0058, 0.22, and 0.84 Gy exposure. Clustering of the 429 regulated transcripts revealed distinct differences between the transcription profiles of different exposures, and only 105 transcripts were regulated in ≥ 2 groups (Fig 2, Table 2). Transcripts regulated in ≥ 2 groups generally had the same direction of regulation and downregulation was more common than upregulation (Table 2). Lastly, very few statistically significant genes had a fold changes between -2 and -1.5, and 1.5 and 2. In all, only 1, 2, 1, 1, 2, and 1 genes in the 0.057, 0.11, 0.17, 0.22, 0.84, and 3.0 Gy groups would be excluded if the fold change cut-off increased from |1.5| to |2|. *Cdkn1a*, a p53 regulated and radiation responsive gene, was statistically significant regulated only in the highest absorbed dose group. When performing validation of microarray data using qPCR, statistically different average gene expression level between irradiated animals and controls was also identified, (Cdkn1a FC: microarray = 5.2; qPCR = 26). The animal-wise correlation between qPCR and microarray measurements for *Cdkn1a* was weak, although the slope is statistically significant different from 0 (Fig 3).

**Table 1:** Regulated transcripts.

Absorbed dose (Gy)	Regulated transcripts		
	Total number	Upregulated	Downregulated
0.0058	98	27	71
0.057	9	3	6
0.11	18	13	6
0.17	102	83	19
0.22	184	53	131
0.50	6	3	3
0.84	57	9	48
3.0	98	28	70

**Table 2:** Shared regulated transcripts.

Transcripts regulated in	Number of transcripts			
	Totally regulated	Upregulated	Downregulated	Up- or downregulated
≥ 1 groups	429	171	236	22
≥ 2 groups	105	13	70	22
≥ 3 groups	20	4	13	3
≥ 4 groups	11	2	7	2
≥ 5 groups	5	1	4	0
≥ 6 groups	2	1	1	0
≥ 7 groups	1	1	1	0
8 groups	0	0	0	0

Up or downregulated transcripts are transcripts not regulated in the same direction when comparing groups, i.e. upregulated in one group, but downregulated in another

### Recurrently regulated genes

**Fig 4:**
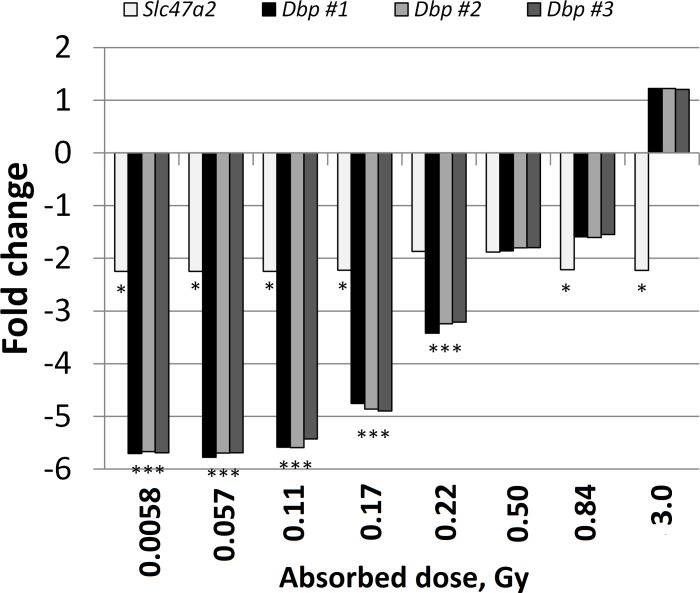
Recurring regulation of Slc47a2 and Dbp. Four of the five transcripts that were regulated after ≥ 5 of 8 different exposures were related to Slc47a2 and Dbp. * indicates regulation, i.e. fold change and Benjamini Hochberg (False Discovery Rate) adjusted p-value cutoffs of 1.5 and 0.01, respectively. Fold change cannot have a value between -1 and 1.

Five transcripts, of which three were associated with the *Dbp* gene and one with the *Slc47a2* gene, were regulated in ≥ 5 groups (Fig 4, Table 3) (the fifth transcript belongs to an uncharacterized protein: RGD1562420). The three *Dbp* transcripts (annotated B in Fig 2) were similarly downregulated after exposure to 0.0058–0.22 Gy, with a monotonous decrease in downregulation with absorbed dose from 0.11 to 0.22 Gy. Changes in *Dbp* gene expression were not statistically significant in response to 0.50–3.0 Gy, although all three *Dbp* transcripts showed similar changes in gene expression levels. Also, regulation of *Dbp* was validated using qPCR, with high consistency with microarray measurements (Fig 3). The transcript associated with *Slc47a2* was consistently downregulated (fold change ~2.2) after 0.0058–0.17, 0.84, and 3.0 Gy exposures (annotated A in Fig 2). RGD1562420 was consistently upregulated (fold change 1.3–2.3 without any monotonous dose-response relationship).

**Table 3:** Recurring regulation of *Slc47a2* and *Dbp*.

Absorbed dose (Gy)	Mean fold change (q-value)			
	*Slc47a2*	*Dbp* #1	*Dbp* #2	*Dbp* #3
0.0058	-2.2 (0.0014)	-5.8 (0.0006)	-5.7 (0.0019)	-5.7 (0.0001)
0.057	-2.2 (0.0086)	-5.6 (0.0001)	-5.6 (0.0001)	-5.4 (0.0001)
0.11	-2.2 (0.0043)	-4.8 (0.0001)	-4.9 (0.0001)	-4.9 (0.0001)
0.17	-1.9 (0.35)	-3.4 (0.0001)	-3.2 (0.0001)	-3.2 (0.0007)
0.22	-1.9 (0.27)	-1.9 (0.99)	-1.8 (1)	-1.8 (0.79)
0.50	-2.2 (0.001)	-1.6 (1)	-1.6 (1)	-1.5 (1)
0.84	-2.2 (0.0001)	1.2 (1)	1.2 (1)	1.2 (1)
3.0	-2.2 (0.0001)	1.2 (0.0001)	1.2 (1)	1.2 (1)

Values are given as fold change and with q-value within parenthesis.

### Upstream regulator analysis

Results from upstream regulator analysis are shown in Table 4. In total, 4, 12, 6, 2, and 2 endogenous upstream regulators were identified in response to 0.058, 0.17, 0.22, 0.84, and 3.0 Gy, respectively. Predicted inhibition of upstream regulators was more common than predicted activation. Upstream regulators related to thyroid function, e.g. TSH, were identified for after 0.0058 and 0.22 Gy. Predicted upstream regulators in the 3.0 Gy group were also found in after 0.17 Gy, while upstream regulators identified in the 0.50 Gy group were not shared with any other group.

**Table 4:** Endogenous upstream regulators identified using IPA.

IA (kBq)	D (Gy)	Upstream regulator	Prediction	Z-value	Involved molecules*
9	0.0058	CREB1	Inhibited	-2.4	CALCB, CHGA, NKX2-1, PAX8, TG, TPO, TSHR
		CTNNB1	Inhibited	-2.2	CDH16, CHGA, DIO1, HHEX, ID4, NKX2-1, PAX8
		NKX2-1	Inhibited	-2.2	SFTPD, SLC5A5, TG, TPO, TSHR
		TSH	Inhibited	-2.2	DIO1, PAX8, SLC5A5, TG, TPO, TSHR
88	0.057	None detected			
170	0.11	None detected			
260	0.17	GATA4	Inhibited	-2.9	CNFN, LOR, MYH6, MYH7, MYL2, MYL4, SPRR1A, SPRR3, TNNC1
		GATA6	Inhibited	-2.6	CNFN, LOR, MYH6, MYH7, SPRR1A, SPRR3, TNNC1
		MYOCD	Inhibited	-2.4	MYH6, MYH7, MYL2, MYL4, TNNC1, TNNI1
		MEF2C	Inhibited	-2.4	MYH6, MYH7, MYL2, MYL4, TNNC1, TNNI1
		MED1	Activated	2.2	LY6D, MYH7, TNNC1, TNNI1, TNNT1
		SRF	Inhibited	-2.2	MYH6, MYH7, MYL2, MYL3, MYL4, SERPINB10, SERPINB2, TNNC1, TNNT1
		STAT5A	Activated	2.2	MYH7, MYL2, TNNC1, TNNI1, TNNT1
		SOX7	Inhibited	-2.0	CNFN, LOR, SPRR1A, SPRR3
		GRB2	Inhibited	-2.0	CNFN, LOR, SPRR1A, SPRR3
		PAX1	Inhibited	-2.0	CNFN, LOR, SPRR1A, SPRR3
		EHF	Activated	2.0	CNFN, KLK5, KLK8, SPRR3
		JUN	Activated	2.0	CSTA, DKK1, LOR, NEFH, SERPINB2, SPRR1A
340	0.22	CREB1	Inhibited	-2.4	CALCB, CHGA, CHGB, FAM46A, HLA-A, MARCKSL1, NKX2-1, PAX8, SCG2, SST, TG, TPO, TSHR
		TSH	Inhibited	-2.3	DIO1, Mt1, PAX8, SLC5A5, TG, TPO, TSHR
		NKX2-1	Inhibited	-2.2	AQP5, FAM3D, HLA-A, LRP2, Serpinb6b, SLC5A5, SOX2, TFF2, TG, TPO, TSHR
		NEUROG3	Inhibited	-2.2	Cd24a, CHGA, NKX2-1, PAX4, SST
		TNF	Activated	2.1	ADAM8, ALDH3A1, AQP5, BCL3, CABP1, DIO1, EMP1, HLA-A, MAN1C1, MARCKSL1, MSLN, Mt1, NKX2-1, PCP4, PTGS1, RGL3, SCN9A, SDC2, SLC5A5, TG, TPO
		PTPRJ	Inhibited	-2.0	SLA, TG, TPO, TSHR
760	0.50	None detected			
1300	0.84	Estrogen receptor	Inhibited	-2.6	CALCB, CDH1, CDH16, CLDN3, CLDN4, DSC2, DSP
		SMARCB1	Inhibited	-2.0	CDH1, Defb1, DSC2, IGFBP2
4700	3.0	SRF	Inhibited	-2.2	ACTC1, ANKRD1, CDKN1A, MYH2, MYH6, MYH7, MYL2, MYL3, SERPINB10, TNNT1
		MEF2C	Inhibited	-2.0	ACTC1, COL2A1, MYH6, MYH7, MYL2, Pln, TNNI1

IA: Injected activity, D: estimated absorbed dose to thyroid. The z-value is an indicator of certainty of activation or inhibition; a high absolute z-value indicates high certainty.

*IPA predicts upstream regulation from human data.

### Functional annotation using Gene Ontology terms

Thirteen transcripts (12 genes) in the present study were annotated Gene Ontology terms related to thyroid gland (Table 5). Downregulation was found in 10/12 genes (*Cpq*, *Dio1*, *Hhex*, *Iyd*, *Nkx2-1*, *Pax8*, *Slc5a5*, *Tg*, *Tpo*, and *Tshr*) and upregulation in 2/12 genes (*Hoxd3* and *Tbx1*) after ^131^I exposure, but most of these changes were only statistically significant in response to 0.0058 and 0.22 Gy (11/12 genes), and to some extent after 0.11 and 0.84 Gy (1/12 and 2/12 genes, respectively). Animal-wise correlation between qPCR and microarray data for *Iyd*, *Pax8*, *Tg*, and *Tpo* served as validation of the microarray measurements (Fig 3). Additionally, due to large variations within the treatment groups, differences in average values in gene expression between irradiated animals and controls were rarely statistically significant (but always showed a trend with similar direction of regulation between qPCR and microarray measurements, S1 Fig).

**Table 5:** Regulated genes related to thyroid function using Gene Ontology terms.

Probe	Gene	Biological process (Gene Ontology)	Fold change (adjusted p-value)															
			9 kBq		88 kBq		170 kBq		260 kBq		340 kBq		760 kBq		1300 kBq		4700 kBq	
A_64_P139744	*Hoxd3*	Thyroid gland development	**3.5**	(0.0096)	1.1	(1)	3.5	(0.065)	1.7	(1)	1.3	(1)	1.1	(1)	1	(1)	1.5	(1)
A_64_P009857	*Tbx1*	Thyroid gland development	1.5	(1)	-1.1	(1)	1.9	(0.72)	1.1	(1)	**2.8**	(0.0002)	-1.2	(1)	1.4	(1)	-1.4	(1)
A_43_P12622	*Cpq*	Thyroid hormone generation	**-3.5**	(0.0046)	-1.6	(1)	-2	(0.61)	-1.6	(1)	**-3.7**	(0.0001)	-1.3	(1)	-2.1	(0.28)	1.1	(1)
A_64_P155193	*Dio1*	Thyroid hormone generation	**-9.8**	(0.0038)	-2.3	(1)	-2.8	(1)	-2.5	(1)	**-13**	(0.0001)	-1.7	(1)	-4.3	(0.23)	-1.2	(1)
A_43_P12437	*Hhex*	Thyroid gland development	**-6.1**	(0.0006)	-2.1	(1)	**-4.6**	(0.0064)	-2.5	(0.6)	**-9.2**	(0.0001)	-2.8	(0.2)	**-4.9**	(0.0022)	-1.1	(1)
A_43_P21810	*Iyd*	Thyroid hormone generation	**-13**	(0.0024)	-2	(1)	-2.9	(1)	-1.5	(1)	**-16**	(0.0003)	-1.4	(1)	-2.8	(1)	-1.2	(1)
A_42_P563276	*Nkx2-1*	Thyroid gland development	**-18**	(0.001)	-2.8	(1)	-4	(0.84)	-2	(1)	**-12**	(0.0002)	-1.9	(1)	-3.2	(0.81)	1.2	(1)
A_64_P097669	*Nkx2-1*	Thyroid gland development	**-12**	(0.0075)	-2.3	(1)	-3.7	(1)	-2	(1)	**-11**	(0.0034)	-1.5	(1)	-3.2	(1)	1	(1)
A_64_P105580	*Pax8*	Positive regulation of thyroid hormone generation, thyroid gland development	**-21**	(0.0001)	-2.8	(1)	-4.3	(0.24)	-2.6	(1)	**-30**	(0.0001)	-1.9	(1)	-6.5	(0.036)	1	(1)
A_44_P115192	*Slc5a5/NIS*	Iodide transport, sodium ion transmembrane transport, sodium ion transport, thyroid hormone generation	**-21**	(0.0001)	-2.3	(1)	-4.3	(0.51)	-2.1	(1)	**-13**	(0.0001)	-2	(1)	-5.7	(0.15)	1.1	(1)
A_64_P128458	*Tg*	Iodide transport, thyroid gland development, thyroid hormone generation, thyroid hormone metabolic process	**-48**	(0.0001)	-2.8	(0.75)	-4.6	(0.027)	-1.2	(1)	**-24**	(0.0001)	-2	(1)	**-5.3**	(0.0074)	1.1	(1)
A_64_P155756	*Tpo*	Thyroid hormone generation	**-69**	(0.0001)	-3.5	(0.87)	-6.1	(0.06)	-2.1	(1)	**-52**	(0.0001)	-2.6	(1)	-6.1	(0.033)	1.3	(1)
A_64_P065852	*Tshr*	Thyroid-stimulating hormone signaling pathway	**-7.5**	(0.0022)	-2.6	(1)	-2.5	(1)	-1.6	(1)	**-4.9**	(0.0096)	-1.7	(1)	-2.3	(0.9)	1.1	(1)

These genes are shown since their regulations were related to thyroid function according to functional annotation of genes using Gene Ontology terms (Biological Processes). Genes with statistically significant changes in mean gene expression (fold change ≥ 1.5, adjusted p-value ≤ 0.01) are shown in bold

### Canonical pathway analysis

IPA canonical pathway analysis revealed an impact on thyroid function (also calcium signaling) as shown in Table 6. Exposure to 0.0058 and 0.22 Gy resulted in regulation of transcripts associated with autoimmune thyroid disease signaling and thyroid hormone biosynthesis. Additionally, an exposure of 0.22 Gy resulted in regulation of transcripts associated with thyroid hormone and thyroamine and iodothyroamine metabolism. Impact on calcium signaling was identified in groups receiving 0.17, 0.50, and 3.0 Gy.

**Table 6:** Effect on diseases and functions based on IPA canonical pathway analysis.

Absorbed dose (Gy)	IPA canonical pathway	p-value	Involved molecules*
0.0058	Autoimmune Thyroid Disease Signaling	0.0004	TSHR, TG, TPO
	Thyroid Hormone Biosynthesis	0.0416	IYD, TPO
0.057			
0.11			
0.17	Calcium Signaling	0.0000	MYH7, TNNC1, MYL4, MYL2, CALML5, TNNI1, MYL3, TNNT1, MYH6
0.22	Autoimmune Thyroid Disease Signaling	0.0004	HLA-A, TSHR, TG, TPO
	Thyroid Hormone Biosynthesis	0.0001	IYD, TPO
	Thyroid Hormone Metabolism I (via Deiodination)	0.0205	DIO1
	Thyronamine and Iodothyronamine Metabolism	0.0205	DIO1
0.50	Calcium Signaling	0.0005	MYH7, MYL2
0.84			
3.0	Calcium Signaling	6.27E-10	MYH3, MYH7, MYH2, MYL2, CALML5, ACTC1, TNNI1, MYL3, TNNT1, MYH6

*IPA predicts involved molecules in the form of human proteins. No canonical pathway related to thyroid function or calcium signaling was statistically significant after 0.057, 0.11, 0.84 Gy.

### Diseases and functions analysis

S2 Table show all diseases and functions related to thyroid function generated using the disease and function analysis within IPA on the microarray data. The transcriptional response to 0.057, 0.22, and 0.84 Gy was related to 52, 42, and 10 diseases and functions, respectively, of which many were linked to thyroid cancer and thyroid hormone homeostasis. The 0.11 and 0.17 Gy groups had only annotation to one disease and function each: thyroid gland development and thyroid gland tumor, respectively.

### TSH and T4 plasma levels

**Fig 5:**
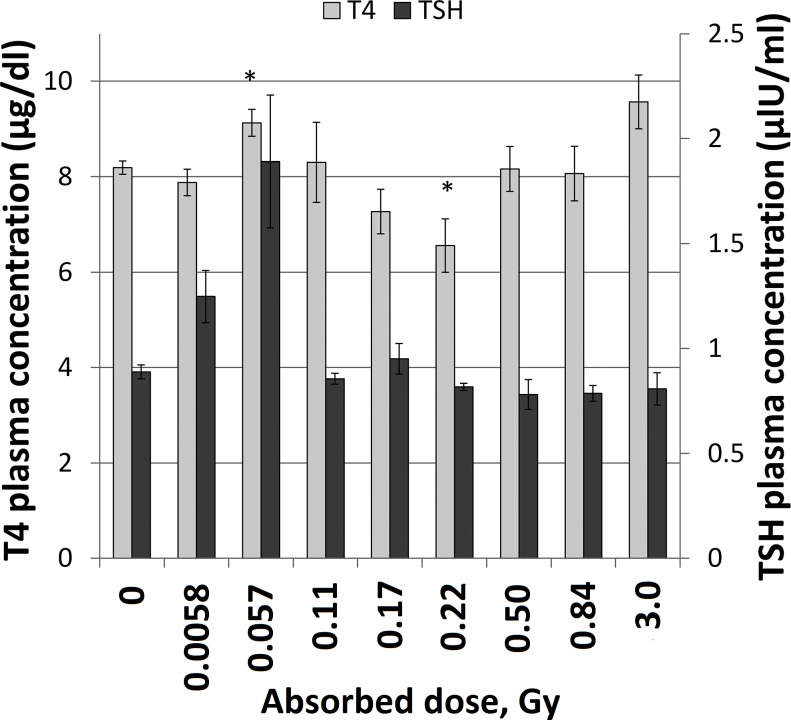
Thyroxine (T4) and thyroid stimulating hormone (TSH) plasma concentration levels. Plasma concentrations of T4 and TSH were measured using ELISA. Bars represent mean values and error bars indicate ± SEM (n = 4). * indicates statistically significant difference between irradiated and control groups (Student’s t-test, p-value ≤ 0.05).

Measured TSH and T4 plasma concentration levels are shown in Fig 5. The response to 0.0058 and 0.057 Gy showed increased TSH plasma concentrations levels, although not with statistical significance. Statistically significant increased and decreased T4 levels were detected after exposure to 0.057 and 0.22 Gy, respectively.

## Discussion

Little is known about which changes in cellular activity occur after ^131^I exposure. Previously, we have investigated transcriptional changes in pooled mouse thyroid tissue after three different exposures to ^131^I [30]. With the present investigation on rats, where eight different absorbed dose levels were used and RNA was extracted from individual rats, we aimed to gain a deeper understanding of the initial biological effects of ^131^I on thyroid tissue that occur on the transcriptional level. Group-specific thyroid absorbed doses were estimated to 0.0058–3 Gy 24 h after administrations of 9–4700 kBq ^131^I. Hence, the absorbed dose levels in the present study are similar to those in the clinical setting and in the case of nuclear fallout, although the dose-rate is likely higher in the present study. Additionally, the present investigation is on the acute response to ^131^I exposure and only one temporal end-point was included. Gene expression is known to fluctuate over time and with dose-rate, and further studies are warranted to understand the effects of ^131^I emitted radiation exposure on temporal changes in rat thyroid[24].

Results from the expression microarrays showed that the number of regulated genes varied considerably between the different groups. This is in agreement with previous reports on gene expression changes in mouse thyroid tissue 24 h after ^131^I and ^211^At administrations although the absorbed dose levels were generally lower in the present study [17, 30]. In the present study, the transcriptional responses observed in rat thyroid tissue after ^131^I exposure involved fewer regulated transcripts, although the majority of regulated transcripts were group/dose-specific (fewer recurrently regulated transcripts) compared with ^131^I exposure in mice [30]. These differences may be in part due to that the RNA microarray analysis was performed using individual (present study) and not pooled tissue samples (previous study on mice) [30]. Another factor is the difference in species.

In the present study, the results indicate a non-monotonic relationship between absorbed dose and number of regulated transcripts. We have previously shown that the thyroid response to lower absorbed dose levels involves regulation of a higher number of genes compared with higher absorbed doses, a phenomenon we still don’t fully understand [17, 23]. We have previously hypothesized that there is a qualitative difference and not only a quantitative difference in response at different absorbed dose levels, something also supported by the present results [17]. This means that the radiation-induced response in vivo differs by type and not only magnitude, and likely reflects the complexity of the radiation-induced response (especially in the in vivo situation). We have also shown a dose-dependence in biological functions (Gene Ontology terms), with an impact on cell communication, and DNA and gene expression integrity at low absorbed dose and cellular integrity and stress responses at higher absorbed dose after ^131^I exposure in mice thyroid [23]. Furthermore, similar findings have been obtained in other tissue types in vivo after exposure to radiohalogens [18, 19]. With this in mind, finding a non-monotonic relationship between the number of regulated genes and absorbed dose may not be that surprising after all.

A dose-response relationship for a biomarker does not need to be linear, but should be monotonic and well-known in order to be useful for biodosimetry. There are different definitions of a biomarker. In the present work, the WHO definition, which is rather inclusive, is used and includes “almost any measurement reflecting an interaction between a biological system and a potential hazard, which may be chemical, physical, or biological” [31]. The transcription factor D site of albumin promoter binding protein isoform 1 (*Dbp*) was recurrently regulated in the present study. The *Dbp* transcripts were regulated similarly after exposure to 0.0058–0.11 Gy but decreased monotonically from 0.11 to 0.22 Gy. This trend continued after 0.50 and 0.84 Gy, although without being statistically significant. This result indicates that *Dbp* might be a biodosimeter for low absorbed doses only, and have some potential for use to distinguish between different absorbed dose levels. The same pattern of monotonic decrease in downregulation with absorbed dose was reported in mouse kidney tissue 8 and 12 months after ^177^Lu-octreotate administration, although at higher absorbed doses compared with the present study (19–45 Gy) [32]. A small increase in *Dbp* expression was identified after 3.0 Gy, although it was not statistically significant. Previous investigations have reported upregulation of *Dbp* 24 and 1–168 h after administrations of ^131^I (0.85–17 Gy) and the alpha emitter ^211^At (0.023–32 Gy) in mouse thyroid tissue [17, 30]. Additionally, *Dbp* was upregulated in mouse kidney 24 h after ^177^Lu-octreotate administrations (0.13–13 Gy) [32]. Altogether, these results suggest that *Dbp* may be a potential biomarker for ^131^I exposure of thyroid, but maybe also for exposure to other radionuclides in other tissues. Taken together, evaluation of variations in *Dbp* gene expression with dose-rate, absorbed dose, and time after administration, and the role of different radiation qualities in different tissues is warranted. Recurring regulation of transporter protein solute carrier family 47 member 2 (*Slc47a2*) was identified in the present study. Radiation-induced changes in expression of *Slc47a2* consisted of similar downregulation in all groups, but not statistically significant after 0.22 and 0.50 Gy. To our knowledge, a relationship between *Slc47a2* expression and ^131^I exposure has not been previously reported. This protein is normally expressed in kidneys in rodents and seems to be involved in the excretion of toxics and drugs, but the action of this protein is probably not fully understood in rodents. Taking into account the results presented in the present paper, it is likely that Slc47a2 can only be used as a binary bio-dosimeter because of the lack of suitable dose-response relationship. Further research is necessary to elucidate the function of *Slc47a2* in the thyroidal response to ^131^I exposure.

Previously, studies have identified late occurring biomarkers of thyroid cancer in the Chernobyl cohorts [33–36]. These studies do not include unirradiated thyroid tissue as controls (as in the present study), but instead compare thyroid cancer and normal thyroid tissues that both were exposed as a result of the Chernobyl incident, which makes direct comparisons with our data somewhat difficult. In one study, expression of eight and six genes were associated with ^131^I exposure [37]. Of these, no specific genes were similarly regulated in the present data, although Ankyrin related genes was regulated after exposure to 0.057, 0.22, 0.84, and 3.0 Gy. We have previously found evidence of decreased Ankyrin (ANK1) levels in mouse thyroid tissue 24 after 32 Gy ^131^I exposure [38]. One additional protein of interest is CLIP2 which has been shown to have a functional role in development of radiation-induced papillary thyroid carcinoma, PTC [35, 36]. *Clip2* was not found regulated in the present study; however, this might be explained by the acute time-point applied here.

An impact on transcription related to thyroid function was identified. In all, 12 genes crucial for thyroid hormone synthesis and secretion were generally downregulated after 0.0058 and 0.22 Gy, and IPA canonical pathway, disease and functions, and upstream regulator analyses associated the transcriptional response to 0.0058 and 0.22 Gy with an impact on thyroid function. Unfortunately, the IPA canonical pathway and disease and function analyses were not able to predict activation or inhibition of pathways or diseases and functions, but if the identified transcriptional downregulation of e.g. *Tg*, *Tpo*, *Slc5a5*/*Nis* and *Tshr* reflect the protein level, thyroid hormone biosynthesis and activity is likely decreased in response to 0.0058 and 0.22 Gy. The qPCR measurements of *Slc5a5*, *Iyd*, *Pax8*, *Tg*, and *Tpo* demonstrated a trend consistent with the microarray data, and 2/3 rats in the 0.0058 Gy group had ≥1000-fold decreased expression levels of all five genes compared with control averages. Additionally, 1 rat in each of the 0.057 and 0.11 Gy groups showed similar expression levels as the two with decreased expression in the 0.0058 Gy group. This trend is consistent with the trend from microarray measurements, where thyroid functions genes generally showed negative fold changes, but with high p-values. In all, these results suggest large variations in thyroid function related gene expression levels between individuals administrated with similar ^131^I activity. Lastly, also consistent with the microarray measurements, an exposure of 3 Gy seems to give no regulatory effect on the *Slc5a5*, *Iyd*, *Pax8*, *Tg*, or *Tpo* genes. We measured decreased plasma T4 levels after exposure to 0.22 Gy, but not after 0.0058 Gy, which at least partly support the transcriptional impact on thyroid function. It is a peculiar pattern with down-regulation of thyroid function related genes almost only in two dose-groups (0.0058 and 0.22 Gy). One explanation for such a behavior can be that at these two absorbed dose levels, a “switch” is hit which results in decreased transcriptional response of thyroid function related genes. We have previously identified a highly dose-specific transcriptional response in thyroid after 1.4 Gy exposure 24 h after ^211^At administration, which supports the existence of such “switches”. Additionally, this response pattern was evident also in extra thyroidal tissues upon ^211^At exposure, suggesting a systemic response after irradiating the thyroid [17, 18]. More research is warranted to determine if the effect on thyroid function is specific to only these two absorbed dose levels (within the dose-interval studied in the present study) or if it is related to biological variation. There have also been reports of thyroid stunning, i.e. where a diagnostic amount of ^131^I diminishes the uptake of subsequent therapeutic administrations of ^131^I [39]. Results in the present article support this phenomenon as explained by ^131^I-induced downregulation of *Slc5a5*/*Nis* and also introduce further complexity since it was only detected at certain absorbed dose-levels [40]. Altogether, these results suggest an impact on thyroid function in the present study, although effects on thyroid function was generally identified in few groups, and with large variation within the groups studied by qPCR. It is unclear if this is a result from biological variation among irradiated animals and if different exposure levels have different effects on thyroid function, 24 h after ^131^I administration.

The canonical pathway analysis also revealed an impact on calcium signaling but it remained unclear whether it reflected effects on normal thyroid function. However, TSH and various calcium-bindings proteins may contribute to calcium-mediated events that could affect thyroid physiology [41]. Further studies are needed to elucidate how, and if, ^131^I exposure affects calcium homeostasis and/or thyroid function. Interestingly, previous reports have demonstrated transcriptional regulation of S100 calcium-binding proteins in mouse thyroid tissue exposed to either ^131^I or ^211^At [17, 30]; however, no S100 calcium binding proteins were regulated in the present study. It is also worth mentioning that downregulation of *Pth* (the parathyroid hormone) was identified in the present study, indicating involvement of the parathyroid glands, located on the surface of the thyroid gland, and the parathyroid glands may have been included in the tissue samples studied. The parathyroid glands have no selective uptake of iodide, but might be irradiated by longer ranged electrons emitted by ^131^I located in the thyroid gland.

## Conclusion

Different amounts of injected ^131^I demonstrated distinct transcriptional responses to ^131^I exposure in rat thyroid. Several exposure groups exhibited a response related to thyroid function, already at very low absorbed doses to thyroid (0.0058 and 0.22 Gy). ^131^I induced changes in T4 plasma levels were also detected in rats, where thyroids were exposed to absorbed doses as low as 0.057 and 0.22 Gy. The absorbed dose levels in the present study are representative for both the clinical use and in the case of nuclear fallout of ^131^I.

## Supporting information

S1 Fig qPCR measurements of Cdkn1a, Dbp, Slc5a5, Iyd, Pax8, Tg, and Tpo. To better see the variation within and between the groups, data is shown as dCT values (calculated as CTaverage of reference genes–CTgene of interest), where a low and high dCT value represent low and high expression, respectively, of the gene of interest compared with the average of reference genes (Tarbp2, Hprt1, and Tbp). The difference in marker denotes statistically significant regulation of that certain gene in a specific treatment group compared with control according to microarray measurements (q-value < 0.01): Cross, statistically significant regulation in microarray; circle, not statistically significant regulation in microarray (the direction of regulation was consistent between qPCR and the microarray measurements). Student’s t-test was used to determine statistical significance between average values of gene expression of irradiated groups and control. P-values are shown for each comparison above the markers.
(TIFF)

S1 Table Log2-ratio values and p-values of all probes. Log2 ratio values and false discovery rate adjusted p-values are shown for each exposure group (Benjamini-Hochberg method).
(XLSX)

S2 Table Diseases and functions analysis generated using IPA. The complete list of all diseases and functions associated with the transcriptional response in the present study generated using IPA.
(XLSX)

S1 File NC3Rs ARRIVE Guidelines Checklist. The ARRIVE Guidelines Checklist Animal Research: Reporting In Vivo Experiments.
(PDF)

## References

[1] Hertz S, Roberts A. Radioactive iodine in the study of thyroid physiology: VII. The use of radioactive iodine therapy in hyperthyroidism. JAMA. 1946;131(2):81–6. 10.1001/jama.1946.02870190005002 21025609

[2] Chapman EM, Evans RD. The treatment of hyperthyroidism with radioactive iodine. JAMA. 1946;131(2):86–91. 10.1001/jama.1946.02870190010003 21025610

[3] Schipper ML, Riese CG, Seitz S, Weber A, Béhé M, Schurrat T, et al Efficacy of 99mTc pertechnetate and 131I radioisotope therapy in sodium/iodide symporter (NIS)-expressing neuroendocrine tumors in vivo. Eur J Nucl Med Mol Imaging. 2007;34(5):638–50. 10.1007/s00259-006-0254-8 17160413

[4] Carrasquillo JA, Chen CC. 131I-MIBG Therapy. Nuclear Oncology: Springer; 2013 p. 691–714.

[5] Bombardieri E, Giammarile F, Aktolun C, Baum RP, Delaloye AB, Maffioli L, et al 131I/123I-metaiodobenzylguanidine (mIBG) scintigraphy: procedure guidelines for tumour imaging. EJNMMI. 2010;37(12):2436–46. 10.1007/s00259-010-1545-7 20644928

[6] Giammarile F, Chiti A, Lassmann M, Brans B, Flux G. EANM procedure guidelines for 131I-meta-iodobenzylguanidine (131I-mIBG) therapy. EJNMMI. 2008;35(5):1039–47. 10.1007/s00259-008-0715-3 18274745

[7] Forssell-Aronsson E, Schüler E, Ahlman H. Advances in the diagnostic imaging of pheochromocytomas. Reports in Medical Imaging. 2011;4:19–37.

[8] Wafelman A, Hoefnagel C, Maes R, Beijnen J. Radiochemical purity, at expiry, and radiochemical stability of iodine-131 labelled meta-iodobenzylguanidine concentrates for intravenous infusion. Nuklearmedizin. 1996;35(4):122–5. 8784866

[9] Mangner TJ, Tobes MC, Wieland DW, Sisson JC, Shapiro B. Metabolism of iodine-131 metaiodobenzylguanidine in patients with metastatic pheochromocytoma. J Nucl Med. 1986;27(1):37–44. 3941363

[10] Brans B, Monsieurs M, Laureys G, Kaufman JM, Thierens H, Dierckx RA. Thyroidal uptake and radiation dose after repetitive I-131-MIBG treatments: influence of potassium iodide for thyroid blocking. Medical and pediatric oncology. 2002;38(1):41–6. 11835235 10.1002/mpo.1261

[11] Annex J. Exposures and effects of the Chernobyl accident Sources and Effects of Ionizing Radiation: The United Nations Scientific Committee on the Effects of Atomic Radiation UNSCEAR 2000:451–566.

[12] Métivier H. Chernobyl: Assessment of Radiological and Health Impacts. Chernobyl: Assessment of Radiological and Health Impacts. 2002.

[13] Akahane K, Yonai S, Fukuda S, Miyahara N, Yasuda H, Iwaoka K, et al The Fukushima Nuclear Power Plant accident and exposures in the environment. The Environmentalist. 2012;32(2):136–43.

[14] Cardis E, Kesminiene A, Ivanov V, Malakhova I, Shibata Y, Khrouch V, et al Risk of thyroid cancer after exposure to 131I in childhood. JNCI. 2005;97(10):724–32. 10.1093/jnci/dji129 15900042

[15] Bonnema SJ, Hegedus L. Radioiodine therapy in benign thyroid diseases: effects, side effects, and factors affecting therapeutic outcome. Endocr Rev. 2012;33(6):920–80. 10.1210/er.2012-1030 22961916

[16] Ron E, Lubin JH, Shore RE, Mabuchi K, Modan B, Pottern LM, et al Thyroid cancer after exposure to external radiation: a pooled analysis of seven studies. Radiation research. 1995;141(3):259–77. 7871153

[17] Rudqvist N, Parris TZ, Schuler E, Helou K, Forssell-Aronsson E. Transcriptional response of BALB/c mouse thyroids following in vivo astatine-211 exposure reveals distinct gene expression profiles. EJNMMI research. 2012;2(1):32 Epub 2012/06/16. PubMed Central PMCID: PMC3489558. 10.1186/2191-219X-2-32 22697397 PMC3489558

[18] Langen B, Rudqvist N, Parris TZ, Schuler E, Helou K, Forssell-Aronsson E. Comparative Analysis of Transcriptional Gene Regulation Indicates Similar Physiologic Response in Mouse Tissues at Low Absorbed Doses from Intravenously Administered 211At. J Nucl Med. 2013;54(6):990–8. 10.2967/jnumed.112.114462 23658216

[19] Schuler E, Parris TZ, Rudqvist N, Helou K, Forssell-Aronsson E. Effects of internal low-dose irradiation from 131I on gene expression in normal tissues in Balb/c mice. EJNMMI research. 2011;1(1):29 Epub 2012/01/05. 10.1186/2191-219X-1-29 22214497 PMC3251037

[20] Schuler E, Rudqvist N, Parris TZ, Langen B, Spetz J, Helou K, et al Time- and dose rate-related effects of internal (177)Lu exposure on gene expression in mouse kidney tissue. Nucl Med Biol. 2014;41(10):825–32. 10.1016/j.nucmedbio.2014.07.010 25156037

[21] Schuler E, Rudqvist N, Parris TZ, Langen B, Helou K, Forssell-Aronsson E. Transcriptional response of kidney tissue after 177Lu-octreotate administration in mice. Nucl Med Biol. 2014;41(3):238–47. 10.1016/j.nucmedbio.2013.12.001 24434014

[22] Langen B, Rudqvist N, Parris TZ, Schüler E, Spetz J, Helou K, et al Transcriptional response in normal mouse tissues after iv 211At administration-response related to absorbed dose, dose rate, and time. EJNMMI Res. 2015;5(1):1–12. 25853007 10.1186/s13550-014-0078-7 PMC4384707

[23] Rudqvist N, Schuler E, Parris TZ, Langen B, Helou K, Forssell-Aronsson E. Dose-specific transcriptional responses in thyroid tissue in mice after (131)I administration. Nucl Med Biol. 2015;42(3):263–8. 10.1016/j.nucmedbio.2014.11.006 25496975

[24] Rudqvist N, Spetz J, Schüler E, Langen B, Parris TZ, Helou K, et al Gene expression signature in mouse thyroid tissue after 131I and 211At exposure. EJNMMI research. 2015;5(1):1–14. 26492889 10.1186/s13550-015-0137-8 PMC4615992

[25] Rudqvist N, Spetz J, Schüler E, Parris TZ, Langen B, Helou K, et al Transcriptional Response in Mouse Thyroid Tissue after 211 At Administration: Effects of Absorbed Dose, Initial Dose-Rate and Time after Administration. PloS one. 2015;10(7):e0131686 10.1371/journal.pone.0131686 26177204 PMC4503762

[26] Spetz J, Rudqvist N, Forssell-Aronsson E. Biodistribution and dosimetry of free 211At, 125I- and 131I- in rats. Cancer Biother Radiopharm. 2013;28(9):657–64. PubMed Central PMCID: PMCPMC3793652. 10.1089/cbr.2013.1483 23789969 PMC3793652

[27] Lance GN, Williams WT. A general theory of classificatory sorting strategies II. Clustering systems. The computer journal. 1967;10(3):271–7.

[28] Amundson SA, Do KT, Fornace AJ Jr. Induction of stress genes by low doses of gamma rays. Radiat Res. 1999;152(3):225–31. 10453082

[29] Rudqvist N, Spetz J, Schuler E, Langen B, Parris TZ, Helou K, et al Gene expression signature in mouse thyroid tissue after (131)I and (211)At exposure. EJNMMI Res. 2015;5(1):59 PubMed Central PMCID: PMCPMC4615992. 10.1186/s13550-015-0137-8 26492889 PMC4615992

[30] Rudqvist N, Schüler E, Parris TZ, Langen B, Helou K, Forssell-Aronsson E. Dose-specific transcriptional responses in thyroid tissue in mice after 131I administration. Nuclear Medicine and Biology. 2014. 10.1016/j.nucmedbio.2014.11.006 25496975

[31] WHO. Biomarkers and risk assessment: concepts and principles. 1993.

[32] Schuler E. Biomarker discovery and assessment for prediction of kidney response after 177Lu-octreotate therapy. PhD thesis, Department of Radiation Physics, Institution of Clinical Sciences, Sahlgrenska Academy, University of Gothenburg. 2014.

[33] Abend M, Pfeiffer RM, Ruf C, Hatch M, Bogdanova TI, Tronko MD, et al Iodine-131 dose dependent gene expression in thyroid cancers and corresponding normal tissues following the Chernobyl accident. PloS one. 2012;7(7):e39103 10.1371/journal.pone.0039103 22848350 PMC3405097

[34] Abend M, Pfeiffer R, Ruf C, Hatch M, Bogdanova T, Tronko M, et al Iodine-131 dose-dependent gene expression: alterations in both normal and tumour thyroid tissues of post-Chernobyl thyroid cancers. British journal of cancer. 2013;109(8):2286–94. 10.1038/bjc.2013.574 24045656 PMC3798970

[35] Selmansberger M, Feuchtinger A, Zurnadzhy L, Michna A, Kaiser J, Abend M, et al CLIP2 as radiation biomarker in papillary thyroid carcinoma. Oncogene. 2014. 10.1038/onc.2014.311 25284583

[36] Selmansberger M, Kaiser JC, Hess J, Güethlin D, Likhtarov I, Shpak V, et al Dose-dependent expression of CLIP2 in post-Chernobyl papillary thyroid carcinomas. Carcinogenesis. 2015:bgv043. 10.1093/carcin/bgv043 PMC4496450 25957251

[37] Abend M, Pfeiffer RM, Ruf C, Hatch M, Bogdanova TI, Tronko MD, et al Iodine-131 dose-dependent gene expression: alterations in both normal and tumour thyroid tissues of post-Chernobyl thyroid cancers. Br J Cancer. 2013. 10.1038/bjc.2013.574 PMC3798970 24045656

[38] Rudqvist N. Radiobiological effects of the thyroid gland: Transcriptomic and proteomic responses to 131I and 211At exposure. PhD thesis, Department of Radiation Physics, Institution of Clinical Sciences, Sahlgrenska Academy, University of Gothenburg. 2015.

[39] Jeevanram RK, Shah DH, Sharma SM, Ganatra RD. Influence of initial large dose on subsequent uptake of therapeutic radioiodine in thyroid cancer patients. International journal of radiation applications and instrumentation Part B, Nuclear medicine and biology. 1986;13(3):277–9. 3771260 10.1016/0883-2897(86)90108-x

[40] Norden MM, Larsson F, Tedelind S, Carlsson T, Lundh C, Forssell-Aronsson E, et al Down-regulation of the sodium/iodide symporter explains 131I-induced thyroid stunning. Cancer Res. 2007;67(15):7512–7. 10.1158/0008-5472.CAN-07-0823 17671222

[41] Lorenz S, Eszlinger M, Paschke R, Aust G, Weick M, Fuhrer D, et al Calcium signaling of thyrocytes is modulated by TSH through calcium binding protein expression. Biochimica et biophysica acta. 2010;1803(3):352–60. 10.1016/j.bbamcr.2010.01.007 20083144

