# Supplementary figures and images for: Transcriptional response to 131I exposure of rat thyroid gland
Source: PLoS One. 2017 Feb 21;12(2):e0171797. doi: 10.1371/journal.pone.0171797 (PMC5319760; doi:10.1371/journal.pone.0171797)

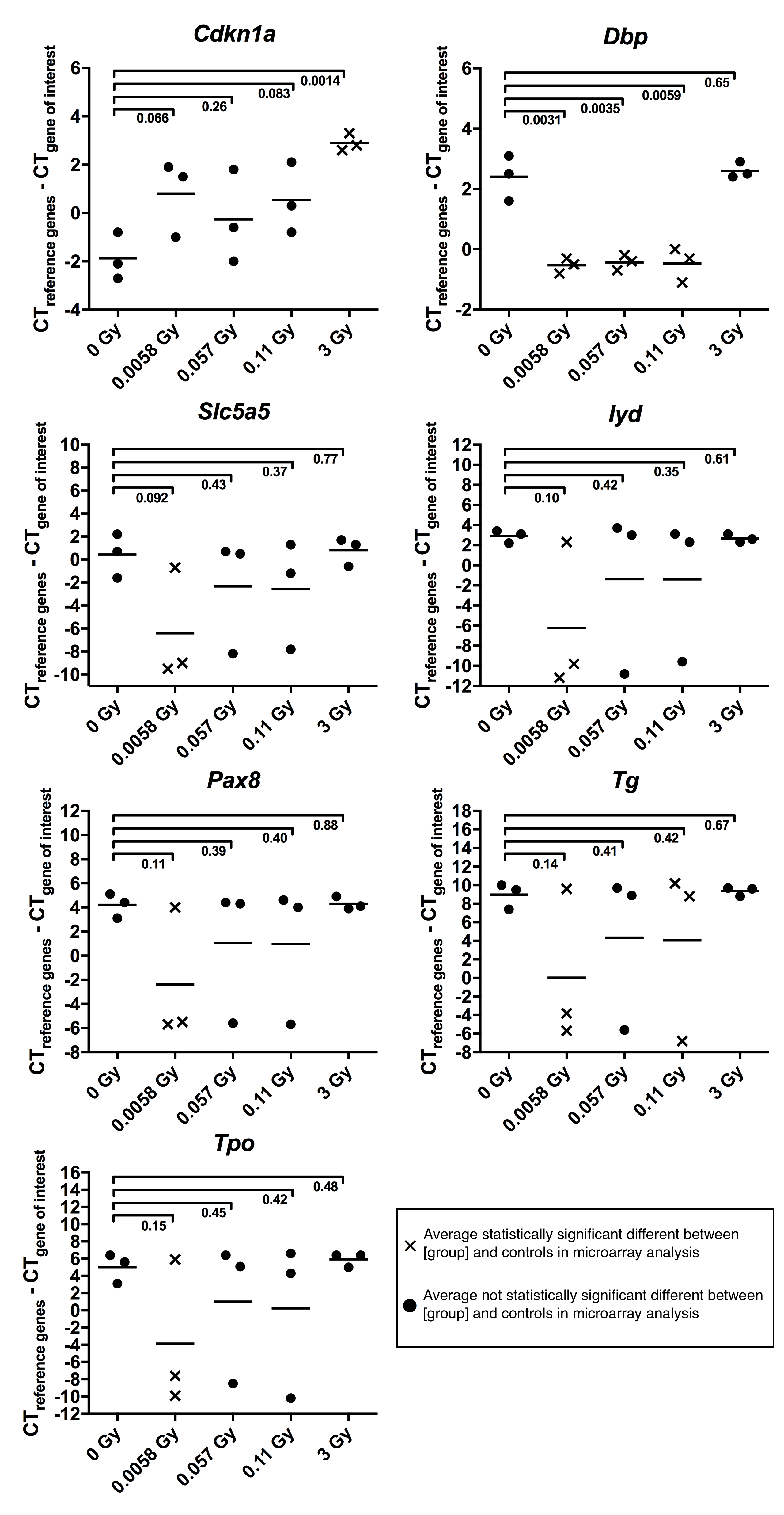

Supplement: S1 Fig — To better see the variation within and between the groups, data is shown as dCT values (calculated as CTaverage of reference genes–CTgene of interest), where a low and high dCT value represent low and high expression, respectively, of the gene of interest compared with the average of reference genes (Tarbp2, Hprt1, and Tbp). The difference in marker denotes statistically significant regulation of that certain gene in a specific treatment group compared with control according to microarray measurements (q-value < 0.01): Cross, statistically significant regulation in microarray; circle, not statistically significant regulation in microarray (the direction of regulation was consistent between qPCR and the microarray measurements). Student’s t-test was used to determine statistical significance between average values of gene expression of irradiated groups and control. P-values are shown for each comparison above the markers. (TIFF) [file pone.0171797.s001.tiff]
